# COVID-19 in Chemical Lung Injury Cases

**DOI:** 10.1017/dmp.2021.13

**Published:** 2021-01-08

**Authors:** Ramezan Jafari, Amin Saburi, Mostafa Ghanei

**Affiliations:** 1 Department of Radiology, Faculty of Medicine, Baqiyatallah University of Medical Sciences, Tehran, Iran; 2 Chemical Injuries Research Center, Systems Biology and Poisonings Institute, Baqiyatallah University of Medical Sciences, Tehran, Iran

**Keywords:** chemical warfare, communicable diseases, infectious disease transmission

The journal recently published a hypothesis review titled, “Are Iranian Sulfur Mustard Gas-Exposed Survivors More Vulnerable to SARS-CoV-2? Some Similarity in Their Pathogenesis.” In this article, possible coronavirus disease (COVID-19) complications in chemical lung injured cases were assessed. Farnoosh et al. aimed to investigate about “the different pathologic aspects of lung injury caused by mustard gas and also the relationship between this damage and the increased susceptibility of Iranian mustard gas exposed survivors to COVID-19.”^1^

The authors first discussed pulmonary complications of sulfur mustard (SM) in clinic and radiologic studies and then pathogenesis of mustard lung, pathogenesis of COVID-19, and the risk of COVID-19 for SM injured cases was discussed. Finally, in conclusion, they predicted that the respiratory and pulmonary clinical manifestations of COVID-19 will be more severe in SM-exposed patients; therefore, COVID-19 is more lethal in SM-exposed individuals.

This review presented compact data from chemically injured lung secondary to SM, which previously presented as mustard lung, and it can be a research model for other industrial and inhalation chemical injuries. There are 2 main pathogenic hypotheses in survivors who inhaled SM: chronic inflammatory process with fluctuation in severity, and oxidative stress-antioxidant imbalance.^2^ Evidence for both hypotheses is interesting and acceptable, with most cases presenting a suitable response to anti-inflammatory or anti-oxidative medications,^3^ but it seems that antioxidant imbalance may be the main basic pathogenesis that triggers the long-term chronic inflammatory process in the lung.^4^

**Figure 1A–D.**
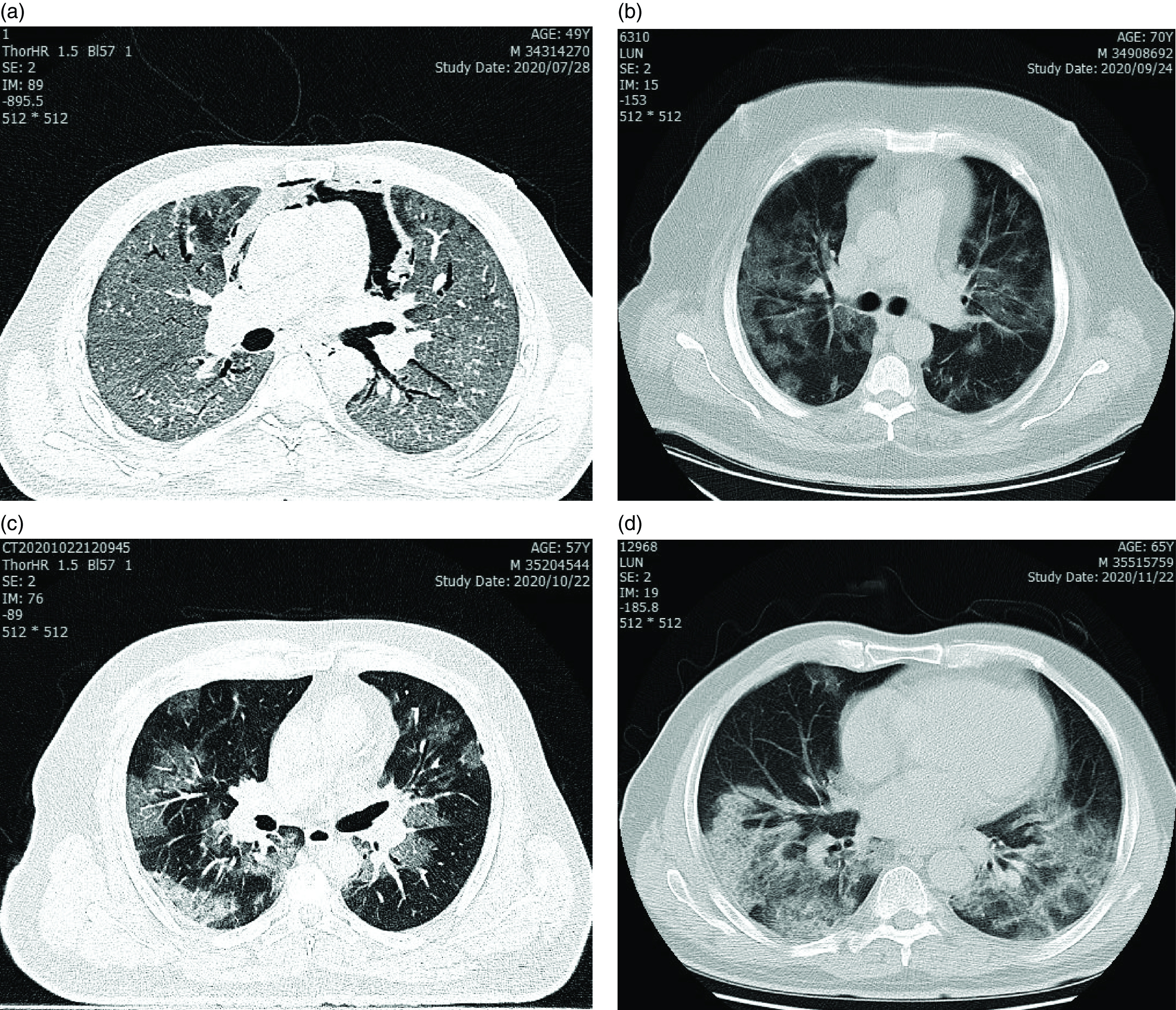
Four cases of SM-injured survivors who were affected with PCR proved COVID-19. In all cases, patchy ground glass opacities and evolving opacities to consolidation were seen, as well as the usual feature of COVID-19 in healthy individuals. Pneumomediastinum as one of the complications of severe cough was observed in the first case (A).

Each mechanism of pathogenesis increases host susceptibility to pulmonary infections like COVID-19 (and virus SARS-CoV-2 that causes COVID-19) infections. Although there is no original research on COVID-19 in SM-injured cases, due to the similarity with chronic obstructive pulmonary disease (COPD) and bronchiolitis obliterans (BO), as the main possible lung pathology in SM injury, we can temporally rely on the COPD case findings. A recent meta-analysis declared that “underlying respiratory diseases, specifically COPD, and smoking were associated with severe COVID-19 outcomes.”^5^ Therefore, the conclusions reached by Farnoosh et al. can be closer to the fact. Here, CT scans of 4 SM pulmonary injury survivors who were affected by COVID-19 will be presented. A CT scan provides the most useful and reliable evidence for the diagnosis of pulmonary COVID-19. Bilateral ground glass opacities and patchy consolidation as the most frequent findings in pulmonary SARS-CoV-2 were observed in these 4 cases and pneumomediastinum was present in 1 case. Two of 4 presented cases were expired due to severity of the diseases (Figure 1, A-B: Cases 1 and 2). Finally, when we carefully looked at the SM lung injuries from pathogenesis to bedside, as well as the pathogenesis of COVID-19, which includes coagulation of proteins and fibrin in combination with debris, infiltration of fibroblasts and other inflammatory cells at interstitium and alveoli, and injury to capillary endothelial cells and also over coagulation process, we concluded that, in these 2 diseases, an intersection/overlap occurs in the accompaniment and type of pulmonary involvement, including the alveoli and small airways (bronchioles), which is likely to exacerbate the disease. Recently, organizing pneumonia (OP) was presented as one of the main possible pathogenesis mechanisms of COVID-19 lung involvement.^6,7^ Therefore, SM-injured case-patients who were previously affected by BO can overlap with new small airway disorders, such as a type of OP, or BOOP. Furthermore, SM-injured survivors are more susceptible for coronary artery disease,^8^ which in itself predisposes the patients to more mortality in SARS-CoV-2 disease.

This feature can accelerate the deterioration and severe phase of the illness and impair the oxygenation cycle. A cohort study on SM-lung-injured survivors, along with healthy controls, is needed to finally prove this vulnerability.
